# Hepatitis B x antigen (HBx) is an important therapeutic target in the pathogenesis of hepatocellular carcinoma

**DOI:** 10.18632/oncotarget.28077

**Published:** 2021-11-23

**Authors:** Arvin Medhat, Alla Arzumanyan, Mark A. Feitelson

**Affiliations:** ^1^Department of Molecular Cell Biology, Azad University, North Unit, Tehran, Iran; ^2^Department of Biology, College of Science and Technology, Temple University, Philadelphia, PA, USA

**Keywords:** chronic liver disease, hepatocellular carcinoma, HBx, functional cure, epigenetic

## Abstract

Hepatitis B virus (HBV) is a human pathogen that has infected an estimated two billion people worldwide. Despite the availability of highly efficacious vaccines, universal screening of the blood supply for virus, and potent direct acting anti-viral drugs, there are more than 250 million carriers of HBV who are at risk for the sequential development of hepatitis, fibrosis, cirrhosis and hepatocellular carcinoma (HCC). More than 800,000 deaths per year are attributed to chronic hepatitis B. Many different therapeutic approaches have been developed to block virus replication, and although effective, none are curative. These treatments have little or no impact upon the portions of integrated HBV DNA, which often encode the virus regulatory protein, HBx. Although given little attention, HBx is an important therapeutic target because it contributes importantly to (a) HBV replication, (b) in protecting infected cells from immune mediated destruction during chronic infection, and (c) in the development of HCC. Thus, the development of therapies targeting HBx, combined with other established therapies, will provide a functional cure that will target virus replication and further reduce or eliminate both the morbidity and mortality associated with chronic liver disease and HCC. Simultaneous targeting of all these characteristics underscores the importance of developing therapies against HBx.

## INTRODUCTION

HBV is a major etiologic agent responsible for the development of chronic liver disease (CLD) which may resolve or progress to HCC. Although there are a variety of treatment options for patients with chronic hepatitis B associated HCC, prognosis and subsequent survival is poor, in part, because diagnosis is often made late, which limits the potential success of available treatments. Since the carrier state and progression of CLD are major risk factors for the development of HCC [1], alternative approaches need to be developed to slow the progression of CLD and reduce the risk of HCC and/or more effectively treat HCC upon diagnosis.

The HBV encoded x antigen, HBx, is the virus contribution to the development of HCC. HBx contributes to pathogenesis in at least three important ways. First, HBx acts epigenetically to promote virus gene expression and replication [2]. Second, HBx helps to protect infected hepatocytes from immune mediated destruction and promotes both cell survival and growth so that the virus will continue to replicate [3]. Both of these roles promote the development and maintenance of chronic infection and the carrier state. Third, many of the properties that promote CLD progression overlap with hallmarks of cancer [4], suggesting that HBx contributes to HCC by epigenetically altering patterns of host gene expression [2], while immune mediated CLD is the host contribution to tumor development [5]. Accordingly, this perspective piece highlights the role of HBx in virus replication and the pathogenesis of HCC, thereby providing strong rationale for pursuing HBx as a therapeutic target of drug development. This also provides a very large therapeutic window, extending from the time of infection when HBx promotes virus gene expression and virus replication to the development of HCC decades later.

### Limitations of prior intervention strategies

An important reason for considering HBx as a therapeutic target has to do with the limitations of current therapeutic approaches. The goal of therapeutic intervention has been to reach a functional cure, defined as sustained off-treatment clearance of hepatitis B surface antigen (HBsAg) particles from blood, which occurs naturally in acute, resolving infections. However, this has not been achieved by any of the treatments currently available for patients with CLD. Initially, interferons (IFNs) and pegylated IFNs, which stimulate anti-viral immunity and block virus transcription and subsequent translation of virus proteins, were developed. Although virus resistance to IFN and pegylated IFN does not appear, these treatments were only effective in clearing HBsAg in only about 10–15% of HBV patients, require frequent intravenous administration, and often have serious side effects [6]. IFN up-regulates the expression of apolipoprotein B mRNA-editing enzyme catalytic polypeptide-like 3G (Apobec3G), which promotes G to A mutations in virus DNA that strongly inhibit HBV replication. However, this activity is suppressed by HBsAg, resulting in diminished IFN efficacy [7]. Although several interferon stimulated genes (Tripartite Motif Containing [TRIM] 31 and TRIM5ɣ) bind to and promote the degradation of HBx, thereby decreasing virus gene expression and replication [8], IFNs are no longer first line therapies for patients with chronic hepatitis B. Instead, a series of potent nucleoside analogs, including tenofovir and entecavir, which are administered orally, and have little toxicity, are now in widespread use. They target the virus polymerase, and greatly reduce virus replication in both short- and long-term applications, but relapse in both virus and CLD occur when treatment is discontinued [6]. Resistance has been demonstrated following long-term treatment with nucleoside analogs, and while mutations conferring resistance have been mapped to the polymerase gene [9], compensating mutations in HBx have also been documented [10]. Even so, only a low frequency of resistance has been observed with most nucleoside analogs. Combination therapy using pegylated IFN and nucleoside analogs did not provide greater benefit (loss of HBsAg) than monotherapy in most studies [6]. One of the reasons for this is that nucleoside analogs inhibit virus replication, but not the transcription of virus mRNAs that encode the other virus proteins. Accordingly, compounds have been sought that inhibit HBV transcription in the hope that they will improve the outcome of anti-viral treatment [11].

Other approaches aimed at preventing or curing infection include the development of inhibitory RNAs that block virus gene expression [12], and inhibitors of HBsAg which block virus infection [13], but these approaches are in early stages of development. For example, surface antigen peptides encoding the virus receptor for infection are being evaluated as decoys to block infection. Small molecules (ezetimibe and cyclosporin) and monoclonal antibodies binding HBsAg epitopes responsible for infection have been developed, but so far, they have reduced but have not blocked infection [14]. Alternatively, phosphorothiolated oligonucleotide polymers targeting the surface antigen gene of HBV have been shown to block the release of HBsAg from infected hepatocytes. Use of this approach in combination with the nucleoside analog, tenofovir and pegylated IFN showed a sustained HBsAg loss in 50% of patients after 48 weeks of follow-up [14]. This suggests that selected combination therapies appear to provide a viable approach toward the development of a functional cure that would reduce the risk of infection, CLD and HCC.

Additional compounds that inhibit or disrupt assembly of virus nucleocapsid particles or lead to the production of core particles that lack virus nucleic acids are also being pursued [15]. The importance of these capsid assembly inhibitors is that HBV nucleic acid replicates within core particles prior to the budding of mature virus particles. When HBV infects cells, the partially double stranded DNA in the virus particle becomes fully double stranded and then appears as a mini-chromosome in the nuclei of infected cells. This mini-chromosome is the template for all virus mRNAs, including a greater than genome length pre-genomic RNA. The latter migrates into the cytoplasm where it becomes packaged along with the virus polymerase into nucleocapsid particles. These nucleocapsids could either acquire an envelope by budding through the endoplasmic reticulum or plasma membrane or could recycle into the nucleus where the newly replicated virus DNA replenishes the pool of covalently closed circular (ccc) DNA that makes up the virus mini-chromosomes [16], which in turn, sustains HBV replication in chronic infection. The problem with current therapies is that they do not eliminate the pool of cccDNA [17] so that the rationale for developing capsid inhibitors (as well as other inhibitors) is to limit the replenishment of cccDNA. Deployment of capsid inhibitors is also expected to slow down the emergence of virus mutants and permit the development of host immune responses that may clear virus replication completely, especially if combined with other modalities. Recent work has shown that such inhibitors are active *in vivo* with low toxicity and that some act synergistically with nucleoside analogs in a preclinical animal model [18]. Capsid inhibitors have also shown a modest decrease in the levels of HBV DNA in chronically infected patients [14]. However, HBx, as the regulatory protein of HBV, binds to the HBV mini-chromosome *in vivo*, thereby *trans*-activating virus gene expression and replication [19], suggesting that targeting HBx may contribute to the elimination of cccDNA in combination with one or more other therapeutic approaches. In fact, early studies showed that elimination of X antgen expression by the introduction of translation stop codons in the related woodchuck hepatitis virus prevented the development of the carrier state, CLD and HCC in experimentally infected animals [20], suggesting that targeting X antigen has potentially far reaching consequences. The finding that HBV DNA from the serum of patients with nonA-nonB hepatitis had variably sized deletions in the HBx gene [21], also suggests that HBx contributes to the establishment and persistence of the carrier state in human populations.

Part of the confusion concerning HBx, which has stunted interest in pursuing it as a therapeutic target, is that it seems to have a large number of reported activities, but it is not clear which activities are important to the biology of HBV [22]. Another limitation is that the structure of native, full-length HBx polypeptide is not known, although there is considerable information on the structure of several functional domains [23]. Structure-function relationships have been proposed based upon computational modeling, circular dichroism, nuclear magnetic resonance, and fluorescence [23]. In addition, technical standards for studying HBx have not been established, so that it difficult to compare results of different assays from various labs [24]. For example, many studies are based on over-expression of HBx in cell lines, and it is not clear whether the results obtained are physiologically relevant. Although HBx interacts with ccc HBV DNA mini-chromosomes *in vivo* to promote virus replication [19], the focus on drug development has been to target ccc HBV DNA, and to date, there are no drugs capable of doing this. Moreover, while most approaches target virus replication, their impact on the pathogenesis of chronic HBV infection remain to be established [14]. In this context, the development of drugs targeting HBx would provide additional tools not only against virus replication, but against CLD and HCC.

### HBx and virus replication

**Figure 1:**
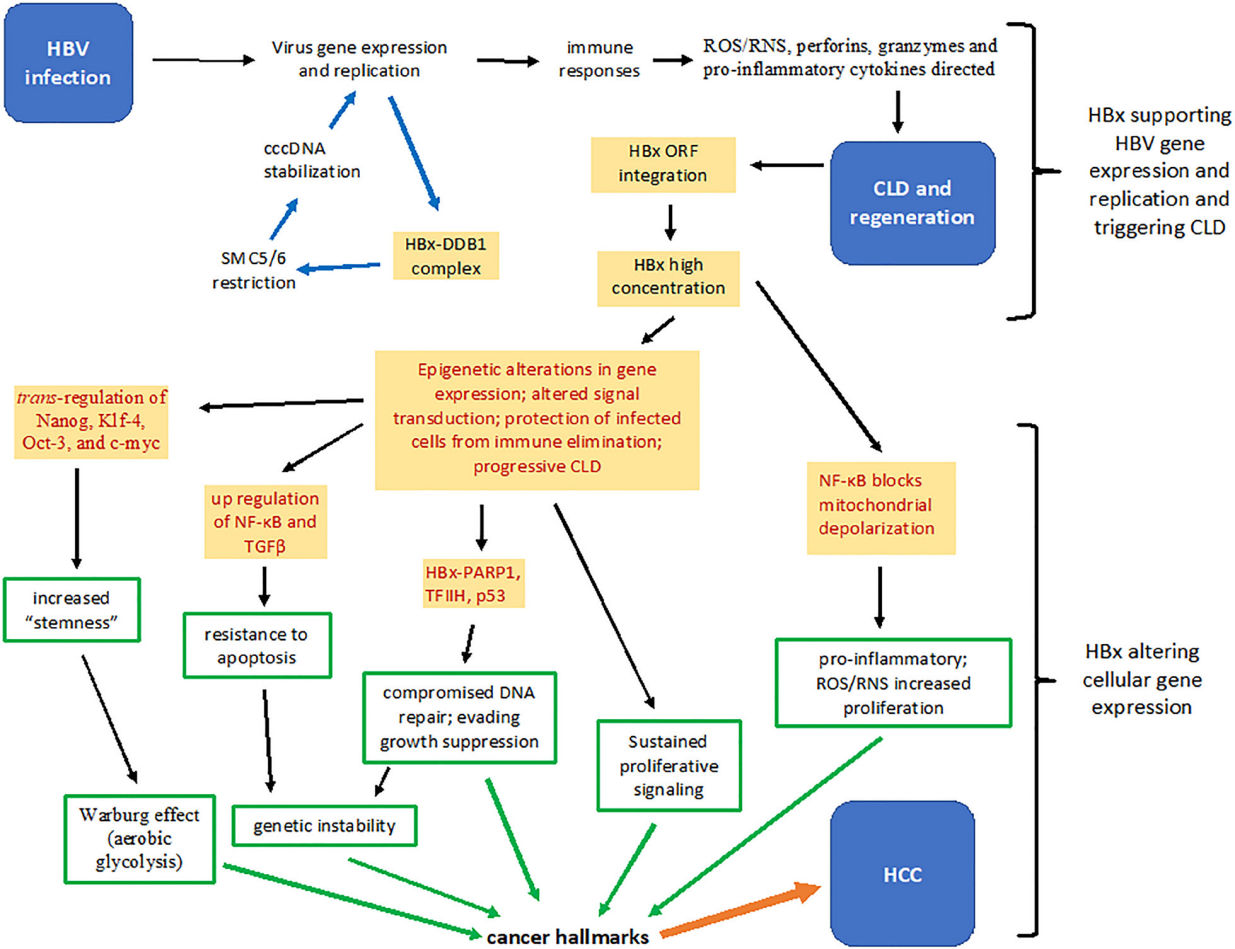
Summary of several HBx functions that contribute to the pathogenesis of chronic liver disease and hepatocellular carcinoma. Upon infection, HBx stimulates virus gene expression and replication by binding a protein complex containing DDB1 and by inactivating the restriction factor SMC5/6 (blue arrows). Hepatocytes replicating virus may trigger immune responses that result in the generation of reactive oxygen and nitrogen species, perforins, granzymes and pro-inflammatory cytokines. Successive bouts of chronic liver disease and hepatocellular regeneration results in increased integration of HBV DNA fragments encoding HBx, which over time accumulates intracellularly. Cytoplasmic and nuclear HBx epigenetically alter patterns of host gene expression that partially protects infected hepatocytes from immune mediated destruction, thereby allowing continued virus replication (red print). Constitutive activation of NF-κB by HBx is both hepatoprotective and pro-inflammatory. HBx promotes the development of “stemness” by up-regulating expression of Nanog, Klf-4, Oct-3 and c-myc. HBx contributes to fibrosis by promoting expression of TGFβ, and inactivates tumor suppressors, such as p53, and inhibits the activity of poly (ADP-ribose) polymerase (PARP1), permitting accumulation of mutations. Mitochondrial associated HBx also alters cellular energetics and the cellular redox state (increases oxidative stress) (red print). Each of these activities contribute to molecular changes that reflect in altered cell behavior (green arrows) that are defined as cancer hallmarks, which in combination, contribute to the development of hepatocellular carcinoma (orange arrow). CLD: chronic liver disease; DDB1: DNA damage-binding protein 1; HBV: hepatitis B virus, ORF: open reading frame; NF-κB: nuclear factor kappa B; RNS: reactive nitrogen species; ROS: reactive oxygen species; TFIIH: transcription factor II H; TGFβ: transforming grow factor beta.

Early studies supported the hypothesis that HBx was associated with virus nucleocapsids [25], that HBx polypeptides in serum correlated with virus replication [26], and that monoclonal antibodies made to core particles isolated from infected liver bind HBx synthetic peptides [27], suggest that HBx is associated with virus particles. This would explain the requirement of HBx for ccc HBV DNA transcription, for the development of viremia, and for the rapid degradation of anti-viral proteins in the cell upon infection [28]. Subsequent studies showed that HBx was associated with the ccc HBV DNA mini-chromosome where it *trans*-activates virus gene expression [28]. HBx’s involvement with DNA repair pathways, particularly its’ binding to the DNA damage-binding protein 1 (DDB1) [29], is critical for virus replication. HBx recruits a DDB1-containing E3 ubiquitin ligase to degrade the host restriction factor SMC5/6, thereby promoting HBV transcription from ccc HBV DNA [30] (Figure 1) HBx also promotes transcription by altering histone post-translational modifications [31], possibly by stimulating histone deacetylases via promoting their recruitment to the mini-chromosome [32] and by acetylation of ccc HBV DNA [33]. HBx stimulates virus gene expression and replication even at low intracellular HBx concentrations, but at high concentrations (where HBx is also made from multiple virus templates integrated into host DNA), HBx also impacts the expression of many host genes [34] (Figure 1). In the latter case, it is proposed that high intracellular concentrations of HBx epigenetically alters hepatocellular differentiation, and the ability of such cells to continue supporting HBV replication. Epigenetic therapy has been proposed as a therapeutic option to target HBx and the activity of ccc HBV DNA, but this approach needs to be explored [35]. In this context, drugs against epigenetic targets in cancer may someday be successfully repurposed for hepatitis B. Inhibition of virus replication in addition to slowing down the pathogenesis of CLD may delay or even prevent the appearance of HCC.

### HBx promotes the progression of CLD and increases the risk for HCC

HBx is a protein binding protein that alters host cell gene expression via epigenetic mechanisms [2]. In doing so, HBx (a) promotes cell viability and proliferation, (b) alters the expression and function of host genes, and (c) accelerates chromosomal instability, (d) contributes to inflammation and fibrogenesis, and (e) malignant transformation (Figure 1). Importantly, even if virus replication complexes are eliminated during chronic infection, HBx production from integrated virus templates may still drive pathogenesis. If so, then targeting HBx will have a dual role in the elimination of both virus and CLD/HCC. In addition, and examination of HBx functions in the development of CLD and HCC will contribute to the rational for the development of different therapeutic approaches that are best suited for different stages of chronic infection.

### Consequences of HBV DNA integration in pathogenesis

Current drugs which target HBV replication have little or no impact upon the fragments of HBV DNA which become integrated into multiple sites within host chromatin during the regenerative process that takes place in the liver following a bout of hepatitis. These integrated HBV DNA sequences often encode the gene expressing HBx [36], and in some cases, up-stream sequences encoding HBsAg and pre-S encoding sequences. Integration events expressing HBx do not occur uniformly throughout the decades of infection [37]. Integration involves multiple sites in different chromosomes, favoring fragile sites [38], and in some cases, integration has been found adjacent to selected cellular genes, such as telomerase reverse transcriptase (TERT) [39], suggesting that HBV may act as an insertional mutagen. In the natural history of infection, an initial immune tolerant phase (stage 1) can develop into a period characterized by immune responses that mediate active hepatitis, repeated integration of HBV DNA sequences into host DNA, and virus reduction or clearance from blood (stage 2). The latter may persist for many years with minimal hepatitis (stage 3), but some carriers undergo reactivation of virus replication with corresponding bouts of CLD and additional virus DNA integration events, which can progress to cirrhosis and HCC (stage 4) [35]. During stage 2, intrahepatic HBx expression is relatively weak and scattered among infected hepatocytes. By stage 4, HBx expression is strong and widespread among infected hepatocytes. At this stage, integration not only results in insertional mutagenesis, but also increased levels of intracellular HBx [40] with both *cis*- and *trans*-activating alterations in host gene expression. Under these conditions, continued virus replication and immune responses targeting virus gene expression may also reveal damage associated molecular patterns (DAMPS) that trigger and maintain auto-aggressive responses that persist even when virus replication becomes undetectable during stage 4. CLD progression may also reflect that HBx appears to protect infected hepatocytes from immune mediated destruction via up-regulation of nuclear factor kappa B (NF-κB, which is hepatoprotective) [41], and that the more integration, the more HBx, and the more impact HBx has on infected cells [42]. HBx also promotes fibrogenesis by strongly up-regulating the expression and signaling of transforming growth factor beta (TGFβ), down-regulating the natural TGFβ inhibitor alpha 2 macroglobulin (α2M) [43, 44] and up-regulating the expression of fibronectin in infected hepatocytes [45]. Elevated TGFβ also promotes angiogenesis, multi-drug resistance, and metastases [46]. HBx stimulation of NF-κB activity also activates the fibronectin promoter, and HBx binding to and inactivating the tumor suppressor, p53, relieves p53 inhibition of the same promoter [46]. In parallel, HBx epigenetically alters cellular properties that constitute many hallmarks of cancer, such as sustained proliferative signaling, resistance to apoptosis/avoiding immune destruction, enhanced genetic instability, and inactivating growth suppression [4]. Importantly, increased intrahepatic expression levels of HBx correlate with the intensity of CLD [40]. Since HBV is non-cytopathic, and pathogenesis is immune mediated, CLD can progress independent of virus replication, associated with accumulation of intrahepatic HBx. In support of this idea, a transgenic mouse model expressing only HBx after birth, with increasing levels of intrahepatic HBx with age, develop CLD leading to HCC within a year [47], indicating that disease progression can occur independent of virus replication. In other HBx transgenic mice, HCC develops in those that express high, sustained intrahepatic HBx, while those expressing low levels of HBx have normal liver histology [48, 49]. If HBV was cytolytic in chronic infection, one would expect a direct correlation between the levels of virus replication and the extent and progression of CLD. In fact, the highest levels of virus replication are often seen among carriers who are asymptomatic and have no liver pathology (stage 1). This is not to say that sustained, high levels of HBV replication do not contribute to the pathogenesis of HCC, since virus replication provides a continuous immunological stimulus that manifests as CLD, which is a known risk factor for HCC [1]. It is just that the majority of chronic carriers remain in stage 1 their entire lives. This involves little hepatocellular turnover and few opportunities for integration of the X ORF into host DNA. It is proposed, therefore, that the epigenetic changes in the patterns of gene expression mediated, in part, by the persistent intrahepatic expression of HBx (from integrated templates) provide the stimulus that drives immune mediated CLD toward HCC in stage 4 of chronic infection [50].

### HBx and pro-inflammatory mediators in CLD pathogenesis

The production of reactive oxygen and nitrogen species, perforins, granzymes and pro-inflammatory cytokines directed against virus infected cells from cell mediated immune responses trigger apoptosis. Cytotoxic T cells also trigger apoptosis via Fas signaling. Cytosolic HBx, stimulated by oxidative stress [51], over-rides these “extrinsic” death signals by blocking selected caspases and by constitutively activating survival and growth pathways [52]. For example, HBx up-regulates the expression of a unique protein (URG7) which blocks tumor necrosis factor alpha mediated apoptosis by activation of phosphoinositol 3-kinase and beta catenin [53]. HBx also impacts upon apoptosis by virtue of its association with mitochondria. HBx triggers mitochondrial membrane depolarization, which would then result in “intrinsic” apoptosis. However, HBx also constitutively activates the transcription factor NF-κB, which blocks apoptosis [54]. HBx also blocks apoptosis by constitutive activation of Wnt/β-catenin, ras/raf/mitogen activated protein kinase (MAPK), activating protein-1 (AP-1) and phosphoinositide 3-kinase (PI3K) signaling pathways [46]. Constitutive activation of NF-κB also blocks anti-Fas mediated apoptosis [55], suggesting that virus infected cells are partially resistant to killing by cytotoxic T lymphocytes. This partial resistance to apoptosis selectively rescues virus infected cells from elimination and provides an important link to a hallmark of cancer (Figure 1). Under these conditions, HBx positive cells regenerate more readily than uninfected hepatocytes, which may explain the strong association between HBx expression and the severity of CLD [40, 56]. This also provides an evolutionary advantage by supporting a reservoir of infected cells that continue to replicate virus and maintain the carrier state even after repeated immune mediated attacks aimed at clearing virus infected cells from the liver [57].

The modulation of apoptosis by HBx appears to be context dependent. As outlined above, HBx could block apoptosis in CLD and via activation of NF-κB. In contrast, when HBx is expressed in normal mouse liver, and such mice are subjected to partial hepatectomy, liver regeneration is largely inhibited [58]. In the latter case, the levels of free radicals, and corresponding HBx expression and activity are low. If HBx promotes cellular survival and growth, then HBx in fully differentiated, quiescent hepatocytes would trigger a cellular response resulting in cell cycle arrest or apoptosis [59]. In a liver undergoing repeated rounds of regeneration, HBx compromises the function of negative growth regulatory pathways and promotes growth stimulatory pathways, so that the outcome of HBx activity depends upon the levels of HBx and the cellular environment in which HBx is expressed [60]. This has a profound impact upon when to treat with HBx targeting therapeutics.

### HBx and “stemness” in HCC pathogenesis

It was discussed above that high intrahepatic concentrations of HBx originating from integrated viral sequences *trans*-regulate the expression of cellular promoters, resulting in altered cellular phenotype. Some of the affected genes encode transcription factors that confer stem-like properties to infected hepatocytes, which include Nanog, Klf-4, Oct-3, and c-myc [61, 62]. Importantly, stem cells are more resistant to apoptosis than parenchymal cells in the same tissue, and are able to grow because ATP production has shifted from oxidative phosphorylation to aerobic glycolysis. This metabolic shift appears to be mediated by HBx [63]. In CLD, the survival and proliferation of cells in cirrhotic nodules, which exist in a hypoxic environment, may result from the “stemness” characteristics conferred onto them by HBx (Figure 1). Recently, it has been shown that hypoxia inducible factors bind ccc HBV DNA and activate the basal core promoter, which promotes HBV transcription and replication [64]. However, HIF may also activate the promoters of HBx (and preS) from the integrated templates encoding these virus products [64], which may stimulate the appearance of “stemness”, cell survival, and oncogenesis. Perhaps this is the reason why there is such strong and consistent staining of HBx in cirrhotic nodules [40, 65]. High levels of HBx originating from multiple integrated templates may contribute to trans-differentiation of hepatocyte or hepatocyte stem cells into biliary tract cells. If so, it may explain the finding that HBx is present in HCC patients with cholangiocarcinoma [40, 65]. If HBx promotes the development of “stemness”, this may contribute to multi-step carcinogenesis years before frank tumors appear, suggesting that HBx is likely to be important therapeutic target for delaying or preventing CLD progression to HCC [3].

### HBx, epigenetics and mutations

While mutations play a central role in carcinogenesis, epigenetic changes in host cell gene expression mediated by HBx may precede and promote the development of mutations. It is proposed that when HBx expression is high in infected liver, it down-regulates the expression of cyclin dependent kinase (CDK) inhibitors and the retinoblastoma gene product, Rb [66], as well as stimulates β-catenin activity [67], but when HBx expression is low or absent, as in HCC nodules, these proteins are often mutated [68] to yield the same functional consequences as their wild type counterparts in HBx expressing cells. This suggests that mutations in these crucial genes are selected for when HBx expression is diminished. This is consistent with the finding that HBx staining is often far lower and sometimes undetectable in HCC nodules compared to surrounding cirrhotic nodules [40, 65]. This implies that HBx triggers epigenetic changes in the liver that are later maintained by genetic mutations in these same genes or pathways in HCC [69]. The reasons for differential expression of HBx during the pathogenesis of CLD may reflect increasing integration of the HBx gene into host DNA over time, but may also reflect post-translational modifications. While HBx is degraded by the ubiquitin-proteasome system, it also interacts with the deubiquitinating enzyme, ubiquitin specific peptidase 15 (Usp15), and also undergoes NEEDylation (a process analogous to ubiquitination), both of which increase HBx half-life [28]. The ser/thr protein kinase Akt1, which is activated by HBx via phosphorylation, and subsequently contributes to hepatocarcinogenesis, also stabilizes HBx by phosphorylation [70].

HBx also promotes the development of mutations by inhibiting DNA repair mechanisms. HBx binds to and inactivates poly (ADP-ribose) polymerase (PARP1), a crucial factor in DNA repair, by inhibiting recruitment of the DNA repair complex to the damaged DNA sites [71]. HBx also interacts with the DNA helicase components of TFIIH, a basal transcriptional factor and an integral component of DNA excision repair [72]. In the human hepatoblastoma line, HepG2, HBx altered the expression of more than two dozen genes involved in DNA repair [73]. HBx also binds to and functionally inactivates wild type p53 [74] so that cell cycle arrest does not occur to permit DNA repair, and apoptosis does not as readily occur, permitting propagation of DNA mutations (Figure 1).

HBx epigenetically alters patterns of host gene expression through stimulation of histone deacetylases 1 and 2, as well as altered expression of DNA methyltransferases 3A and 3B [75]. Genes encoding the cyclin dependent kinase (CDK) inhibitors p14^ARF^, p15^INK4B^, and p16^INK4A^, as well as the Rb gene had significantly higher methylation levels in HBV associated HCC compared to non-virus associated HCC [76]. These findings are consistent with the growth promoting properties of HBx *in vitro* and its’ oncogenic properties in HBx transgenic mice. Thus, targeting HBx activity is expected to potentially decrease the accumulation of epigenetic changes and genetic instability/mutations in the infected cell [62], thereby potentially reducing the risk of multi-step hepatocarcinogenesis (Figure 1).

### HBx alters expression of miRNAs

HBx also impacts host gene expression by regulating the expression of multiple miRNAs. Differential miRNA expression alters the expression and activity of gene products that mediate various signaling pathways in the cell. For example, up-regulated miR-155 promotes β-catenin expression which, in turn, up-regulates c-myc [77]. Up-regulated miR-155 also inhibits the suppressor of cytokine signaling 1 (SOCS1), and down-regulates Janus kinase/signal transducer and activator of transcription (JAK/STAT) signaling [78]. The latter promotes inflammation, and reduces expression of the tumor suppressor, p21^WAF1/CIP1^, resulting in cell proliferation. HBx-upregulated miR-155 also represses HBV replication by modulating CCAAT/enhancer-binding protein (C/EBP) protein that activates the enhancer II/basal core promoter of the virus [79].

HBx-up-regulation of the miR-17-92 family stimulates cell proliferation by repressing the cyclin dependent kinase inhibitors p21, p27, and p57 [80]. HBx-induced miR-181a is anti-apoptotic by repressing FAS/ATG5 to promote cell survival and also promotes the development of “stem-like” features in hepatocytes, which contribute to hepatocarcinogenesis [61]. HBx mediated alterations in miR expression patterns also impact upon innate immune responses to the virus, and combined with the packaging of miRNAs into extracellular vesicles, may also result in the suppression of anti-viral cellular immune responses that serve to promote chronic infection. Thus, altered miRNA expression in infected hepatocytes may alter the function of multiple cell types in both innate and adaptive immunity [81]. These features underscore both the multiple functions of HBx in the infected cell and provide additional rationale for pursuing HBx as a therapeutic target.

### Can targeting HBx contribute to a functional cure?

Efforts of the past few years have aimed at a “functional cure” for HBV, which is defined by the clearance of HBsAg from the blood. Given the persistence of integrated HBV DNA templates expressing virus proteins, and the difficulty in eliminating ccc HBV DNA, complete virus clearance has not been possible. This point is important because high levels of virus replication (with persistence of ccc HBV DNA) in patients with CLD contribute to the development of HCC [82]. In the absence of small molecules that target ccc HBV DNA, gene editing has been used in attempts to completely eliminate ccc HBV DNA, but HBV has a 10-fold higher mutation rate than other DNA viruses because it replicates via reverse transcription, which would lead to the appearance of resistance under therapeutic selective pressure. Targeting all HBV subtypes by gene editing would also be challenging due to about 8% sequence divergence. In addition, since gene editing results in double stranded breaks, this may promote integration of HBV DNA into host chromatin, which may increase the risk of HCC [28]. To avoid these limitations, attempts at therapeutic vaccination against HBsAg and/or providing agonists of toll-like receptor signaling in stage 2 of chronic infection have been performed to boost anti-viral immune responses, but the outcomes have not been consistent [83]. Therapeutic vaccination against HBx has been evaluated in one preclinical model, which provides proof-of-principle that targeting HBx strongly reduces HBV gene expression and replication *in vivo* [84]. Since HBx stimulates virus gene expression and replication, it is possible that such vaccination early on in chronic infection could help to clear virus and reduce the risk of CLD and the development of HCC. However, it is also possible that therapeutic vaccination against HBx (or other virus antigens) may exacerbate CLD in an already damaged liver if virus antigen expression in the liver is widespread, which may increase the risk of liver failure. However, a transient flare of CLD is often observed in carriers seroconverting from HBe (high HBV replication) to anti-HBe (low HBV replication) during natural infection, suggesting that therapeutic vaccination against HBx may be a viable approach to investigate. An important question is whether the generally immunologically tolerant environment of the liver will limit the efficacy of any therapeutic vaccination attempts. Alternatively, transcriptional control of ccc HBV DNA expression may be achieved by targeting HBx or core protein, both of which have been implicated in ccc HBV DNA stability and/or expression [14, 85]. By stage 4 chronic infection, clearance of virus may not be enough to achieve a real cure, because HBx made from numerous integrated HBV DNA sequences is probably at high enough levels to alter both the patterns of host gene expression and the corresponding phenotype of the infected cells independent of virus replication. In HCC nodules, HBx expression is often diminished or may be undetectable compared to adjacent non-tumor liver [40, 65], suggesting that once HBx rewires the patterns of host gene expression, either epigenetically and/or by promoting the development of mutations, its’ expression is no longer selected for during tumor progression. Interestingly, a number of molecules and signaling pathways targeted by HBx in the chronically infected liver are found to be mutated in tumor nodules where HBx expression is diminished or absent [67]. Thus, inhibiting HBx is likely to provide a more complete definition of “functional cure,” and if so, then HBx may be an important therapeutic target in CLD and in delaying or preventing the onset of HCC [3, 50].

### Progress and limitations in targeting HBx

The discussion above strongly suggests that HBx is likely to be an important therapeutic target throughout the course of infection. However, there is no 3-dimensional structure for full length HBx, and it lacks functional motifs with characterized structural information from other proteins, so it has not been possible to use computer aided rational drug design [35]. However, the recent report of the crystal structure of an HBx peptide bound to the BH3 domain of the anti-apoptotic mitochondrial protein, Bcl-xL provides the potential of eventually creating molecules that would disrupt this interaction and be useful by promoting apoptosis [86]. Using a different approach, an HBx monoclonal antibody conjugated to the cell penetrating tat protein was effective in significantly suppressing HBV transcription, replication, and protein production both *in vitro* and *in vivo* [87]. This approach could be useful in combination with nucleoside analogs and/or capsid inhibitors to eliminate HBV replication and render ccc HBV DNA inactive, so that over time, the pool of ccc HBV DNA would become undetectable as well, and a real cure could be achieved. Although clinical trials targeting HBx by these or other approaches have not been conducted [88], administration of monoclonal anti-HBx appears to have anti-tumor activity in HCC bearing mice and patients [89]. An HBx-based therapeutic vaccine has also been shown to clear HBsAg and HBV DNA from carrier mice [90]. HBx specific siRNAs have also been shown to suppress HBV replication in cell lines and in mice [91], suggesting that HBx is likely to be an important therapeutic target. Alternatively, recent work has shown that nitazoxanide (repurposed from its use against protozoan enteritis) prevents the binding of HBx to DDB1 [92]. Given that the HBx/DDB1 complex promotes transcription of ccc HBV DNA by degrading the cell encoded structural maintenance of chromosome complex SMC5/6, the latter of which is a host anti-viral protein, treatment with nitazoxanide may help to restore host anti-viral activity [92]. Alternatively, dicoumarol, a competitive NADPH quinone oxidoreductase (NQO1) inhibitor, was shown to block HBx expression by promoting HBx proteasomal degradation, thereby suppressing ccc HBV DNA transcription [93]. The natural products ursolic acid oleanolic acid also appear to have activity against HBx mediated tumorigenesis, but require further study.

## FUTURE PROSPECTS

Accumulating evidence suggests that HBx contributes to virus replication, protects infected hepatocytes against immune elimination, and contributes to multi-step hepatocarcinogenesis [2, 14] (Figure 1). The fact that HBx alters patterns of gene expression epigenetically, suggests that compounds made in the body or synthetically prepared against targets of the epigenetic machinery altered by HBx would help to lower the risk for the progression of CLD to HCC by re-establishing hepatocellular homeostasis. Further, re-establishment of immunological homeostasis will reduce the progression of CLD to HCC. Decreased inflammation will also reduce the expression and activity of HBx, the production of free radicals, and the appearance of mutations. Decreased levels of HBx will also promote genome integrity, in that Rb, p53 and DNA repair mechanisms will no longer be inhibited. Compounds that promote hepatocyte differentiation in the place of stemness will also lower the risk of hepatocarcinogenesis. Thus, therapeutically targeting HBx may target both virus and disease in a way not possible by other approaches. While there are promising compounds being developed against HBx, as discussed above, most are against virus replication.

Importantly, chronic inflammatory diseases are often characterized by alterations in the composition of the gut microbiota, referred to as dysbiosis, which is accompanied by a “leaky gut” in which bacteria and bacterial metabolites contribute to hepatitis via portal vein transit [94]. Studies have already documented dysbiosis among HBV carriers with liver cirrhosis [95, 96] and HCC [97]. Dysbiosis was often characterized by increased levels of lipopolysaccharide resulting from an overgrowth of gram-negative bacteria, and a decrease of bacteria producing anti-inflammatory compounds such as short chain fatty acids. In fact, feeding HBx transgenic mice short chain fatty acids (SCFAs) three months prior to the appearance of dysplasia or HCC inhibited the development of these lesions in about half the mice, suggesting a cause and effect relationship [98]. Proteomics analysis using liver samples from these mice showed that SCFAs inhibited many pathways that contribute to hallmarks of cancer [98, 99]. There are also a number of comorbidities associated with chronic HBV, including autoimmune diseases and other inflammation associated cancers [100]. Given that gut bacteria make both pro- and anti-inflammatory metabolites that help to maintain immunological homeostasis via immuno-regulation, treatment resulting in the resolution of dysbiosis may mitigate CLD and its progression to HCC. This would potentially provide an epigenetic approach for reversing the effects of HBx in the liver and generating a functional cure characterized by little or no virus replication, amelioration of CLD, and either delayed onset or prevention of HCC.
